# CODON—Software to manual curation of prokaryotic genomes

**DOI:** 10.1371/journal.pcbi.1008797

**Published:** 2021-03-31

**Authors:** Bruno Merlin, Jorianne Thyeska Castro Alves, Pablo Henrique Caracciolo Gomes de Sá, Mônica Silva de Oliveira, Larissa Maranhão Dias, Gislenne da Silva Moia, Victória Cardoso dos Santos, Adonney Allan de Oliveira Veras

**Affiliations:** 1 Postgraduate Program in Applied Computing, Federal University of Pará campus Tucuruí (CAMTUC-UFPA), Pará, Brazil; 2 State University of Pará (UEPA) campus Marabá, Pará, Brazil; 3 Federal Rural University of Amazonia Campus Tomé-Açu (UFRA), Pará, Brazil; 4 State University of Pará (UEPA) campus Santarém, Pará, Brazil; 5 Faculty of Computing Engineering, Federal University of Pará campus Tucuruí (FECOMP-CAMTUC-UFPA), Pará, Brazil; Johns Hopkins University, UNITED STATES

## Abstract

Genome annotation conceptually consists of inferring and assigning biological information to gene products. Over the years, numerous pipelines and computational tools have been developed aiming to automate this task and assist researchers in gaining knowledge about target genes of study. However, even with these technological advances, manual annotation or manual curation is necessary, where the information attributed to the gene products is verified and enriched. Despite being called the gold standard process for depositing data in a biological database, the task of manual curation requires significant time and effort from researchers who sometimes have to parse through numerous products in various public databases. To assist with this problem, we present CODON, a tool for manual curation of genomic data, capable of performing the prediction and annotation process. This software makes use of a finite state machine in the prediction process and automatically annotates products based on information obtained from the Uniprot database. CODON is equipped with a simple and intuitive graphic interface that assists on manual curation, enabling the user to decide about the analysis based on information as to identity, length of the alignment, and name of the organism in which the product obtained a match. Further, visual analysis of all matches found in the database is possible, impacting significantly in the curation task considering that the user has at his disposal all the information available for a given product. An analysis performed on eleven organisms was used to test the efficiency of this tool by comparing the results of prediction and annotation through CODON to ones from the NCBI and RAST platforms.

## Author summary

The accuracy of genome annotation is directly impacted by the manual curation step since complementary information is added to gene products. However, this process takes time and requires specialized labor, since there is a need to consult external databases to check the information of the products inferred in the automated process. We present the CODON software, which allows this process to be dynamic and significantly reduce the total work, the user responsible for conducting manual curation will be able to check the annotation of each product directly on the screen of his computer, without the need to search for the similarity of each ORF in the external databases, it is possible to change the ORF annotation based on the information displayed on the screen, for example, percentage of identity and alignment length match. The results show that CODON is an efficient tool for manual curation, beyond prediction and annotation. In addition to providing the user with access to highly accurate database information, producing a result with more gene acronyms, metabolic pathway information, and Gene Ontology terms.

This is a *PLOS Computational Biology* Software paper.

## Introduction

The advent of DNA sequencing platforms provided a great advance in the deposit of biological information in public databases. This has driven the development of sophisticated algorithms to perform various processes, for instance, to assemble the genomes of organisms sequenced in these platforms. However, knowing the genetic content of an organism is only possible after the annotation process, which consists of inferring structural and functional biological information to genomic sequences [1].

To assist in this task, several tools have been developed. For example, Rapid Annotation using Subsystem Technology (RAST) is a web-based platform for the annotation bacterial and archaeal genomes [2], Prokka is a service for annotating prokaryotic genomes online and in command lines available only for Linux operating systems [1], and National Center for Biotechnology Information (NCBI) also provides an automatic annotation pipeline for prokaryotic genomes (https://www.ncbi.nlm.nih.gov/genome/annotation_prok/).

Despite the variety of computational tools for genome annotation and the associated reduction of annotation time, the automated process still presents several errors: prediction of open reading frame (ORF) errors like incorrect assignments of start or stop codons, unpredicted genes, union or breaking of genes, and the inference of incomplete or even incorrect information [3].

Thus, after the automatic annotation process, it is necessary to perform manual curation during which the information assigned through the previous step is verified. Manual curation is done with the aid of tools such as the Artemis genome browser [4] and searches for similar sequences in public databases such as Uniprot [5], SWISS-PROT [6] and NCBI (https://www.ncbi.nlm.nih.gov/).

The manual curation process is considered difficult and labor-intensive by researchers. A high-quality annotation is considered the gold standard for deposit in public databases as avoiding the propagation of errors is desired. Indeed, performing manual curation requires significant time that is directly related to the number of gene products in the organism under study [3].

In order to assist in the manual curation process, we present the CODON software, a tool that performs the prediction and annotation processes or improves annotation from other predictions and annotation systems and enables manual curation. In this tool, the researcher has the ability to make decisions regarding ORF adjustments and to add biological information through a simple and intuitive graphic interface.

### Design and implementation

**Fig 1:**
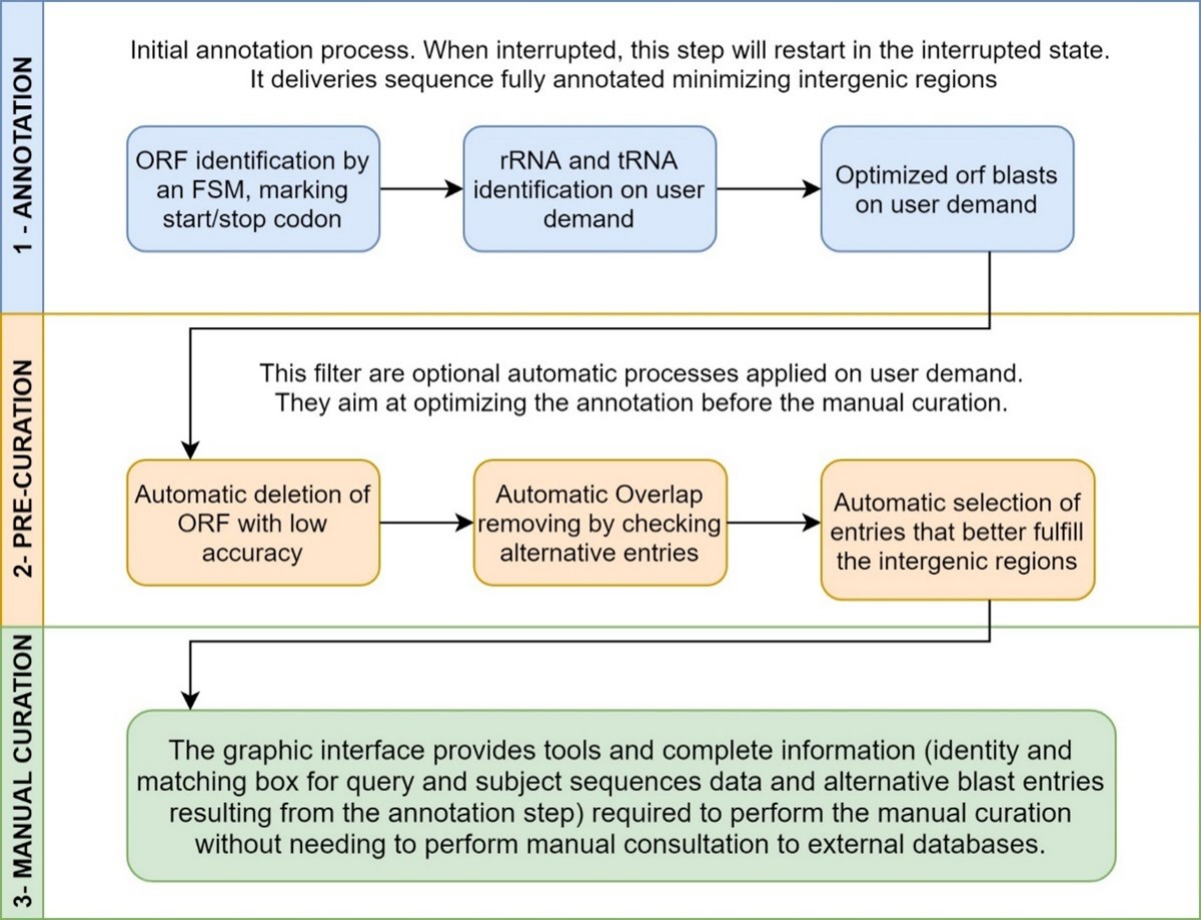
CODON pipeline. Tasks to Complete Annotation From Genome FASTA file.

**Fig 2:**
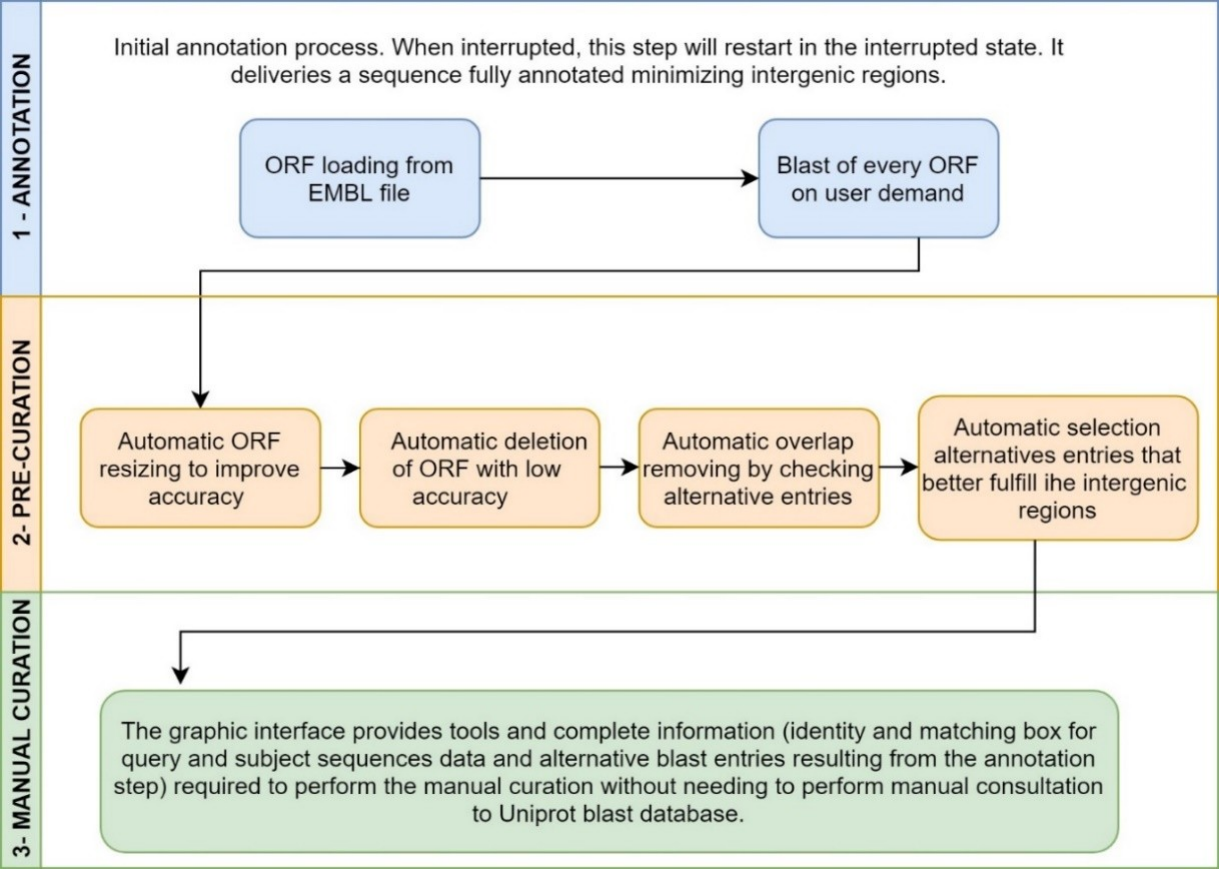
CODON pipeline. Tasks to Re-Annotation From EMBL file.

The annotation process (Fig 1) applied in CODON consists in marking the candidate ORFs using a final state machine, and comparing them to the UNIPROT [5] database. A pre-curation stage enables the user to apply filters in order to improve the resulting annotation. At last, CODON enables the user to finalize the process by a manual curation. CODON accepts a FASTA file as a minimum required input to perform both prediction and annotation process, where the users can customize several parameters (Fig 1). CODON is also able to perform a re-annotation from a former prediction, provide as EMBL or GB file, by another prediction tool such as, RAST for instance (Fig 2).

The ORF annotation or re-annotation process is done by extracting the information obtained within the search and looking for similarities in the UNIPROT database. These data are used in the construction of information sets for each ORF. For every search result (blast entry), the set contains the sequence in amino acids, the name of the identified product, GO (Gene Ontology) term together the ID and the description of each identified term, EC number lists the IDs of metabolic pathways obtained via KEGG, gene acronym is applied if available, and finally the coordinates of the beginning and end of each product.

The major steps of manual annotation are summarized as follows:

- Every product identified and annotated will be shown on the graphical interface.
- For each product, a list of the best hits obtained by Blast is shown. With information on product ID, Identity, Gene, GO Term, EC Number, a graphic alignment scheme similar to that of UniProt, among others.
- Through the graphical interface the user can choose which of the hits on the list he would like to use in the annotation. The information for the chosen hit is automatically incorporated in the selected ORF.
- Overlap problems are marked and can be solved using one of the options: resizing an ORF, removing an ORF or selecting another entry for the ORF
- It is also possible to adjust the length of the products. Depending on the user’s choice, CODON performs this adjustment automatically or manually.
- After the manual curation process is completed, the annotation is exported as an EMBL file.

### The CODON tool

CODON was developed using the JAVA programming language (http://www.oracle.com) and the Swing library was used to develop the graphical interface.

A final state machine based on the genetic code [7] was used to identify the ORFs and mark the start and stop codon coordinates. After this stage, each sequence is submitted to a search for similarities in the UniProtKB database, a database with manually cured, highly accurate information. The database was accessed using UniProtJAPI, Uniprot’s Java API [8].

### Manual curation

CODON allows manual curation through the graphical interface. For every product identified and annotated, there is a list of the top hits obtained from blasting the ORF against the database. Thus, after the analysis, the user can choose to use the information from any of the hits listed.

In the manual curation stage, it is possible to adjust the length of the product based on the size of the product deposited in the database. The tool allows automatic and manual adjustment performed by the user after selecting a hit directly on the CODON interface. This option is also used in the manual solving of product overlaps.

### Prediction and annotation of tRNAs and rRNAs

The prediction and annotation of the tRNAs and rRNAs are performed that recognizes the sequence regions matching with the tRNA sequences stored in the tRNAScan [9] database (http://gtrnadb2009.ucsc.edu/download.html) and RNA sequences stored in the RNAmmer [10] database (http://www.cbs.dtu.dk/services/RNAmmer/).

### Algorithms and metrics

#### Initial ORF delimitation

The prediction process begins with an initial delimitation of start and stop codons based on the genetic code for every candidate ORF. ORFs with a size lower than 60 bases are ignored since lower values become unspecified to search for similarity. However, valine and leucine were added as candidate start codons like in Artemis [4].

The first methionine, valine or leucine codon indicates a new ORF which is terminated when a stop codon is encountered. If an ORF begins by a valine or leucine and the first methionine is located between the start and the stop codons, and if the distance between the methionine and the stop codon is up to 60 bases, then the start of the ORF is replaced by the position of this first methionine. As a consequence, when possible, a candidate ORF starts with a Methionine.

It is worth mentioning that there is no guarantee that the selected start codon is the correct one, the start codon may be updated later during the annotation process. This ORF identification process is performed simultaneously in the 6 frames, in just one execution by a finite state machine.

#### ORF blasting

The nucleotide sequence of the previously identified ORFs are blasted against the UNIPROT database through the API UniprotJAPI [8]. The blast retrieves a set of products registered in the database where the amino acid sequence (the subject) matches partially or completely with the ORF query. For each entry, CODON stores: (i) the entry ID; (ii) the product name; (iii) the product ID; (iv) the gene name if a gene is associated to the entry; (v) the subject amino acid sequence; (vi) the coordinates of the query subsequence (startQuerySeq and endQuerySeq) and subject subsequence (startMatchSeq and endMatchSeq) that have a level of similarity; and (vii) the percentage of similarity both subsequences (identity); (viii) the organism associated to the product; (ix) the GO terms; and (x) the pathway KEGG.

**Fig 3:**
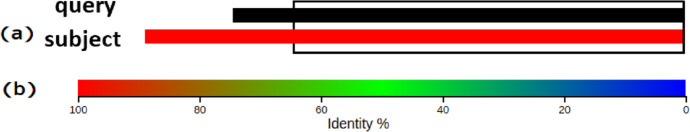
Entries CODON. (a) Graphical blast results for the entries on CODON, (b) Show the color scale used on analysis, query and subject.

The graphical interface of CODON shows the blast results for the entries similar to how they are represented on the UNIPROT website. The query is represented by a dark bar and the subject by a colored one (Fig 3A). An unfilled rectangle highlights the query and subject subsequences that match together.

The subject color depends on the identity value. It is a gradient between RED (100%) and GREEN (50%) when the identity is above 50%, and between GREEN (50%) and BLUE (0%) when the identity is below 50% (Fig 3B).

#### Accuracy

The Accuracy metric (ACC) is used to compare entries retrieve by CODON software. It measures how a UNIPROT entry and an ORF fit together taking into account not only the identity but also the quantity of amino acid for both subject and query matching together. It is calculated using the ORF coordinates and data retrieved from the blast:

- OL: Length of the ORF
- ID: Identity, resulting from the query blast
- SL: Subject length, resulting from the query blast
- ML: Match length, resulting from the query blast (subsequence of the query and the subject that match according to the blast), then ML = endQuerySeq–startQuerySeq = endMatchSeq–startMatchSeq, ML < = SL and ML < = OL.

ACC=ID*(ML/SL)*(ML/OL)=id*ML*ML/(SL*OL) (1)

When ML = SL = OL, which means that the query and the subject match on their whole strength, ACC = ID.

#### Levenshtein distance and similarity score

To compare the distance between two amino acid sequences, CODON uses the Levenshtein distance [11] that scores the difference between two strings. The Levenshtein distance (D) measures how many transformations (character substitutions, insertions or deletions) are necessary to transform one string into another. The Levenshtein distance can be calculated for two strings having different lengths, whereas CODON algorithms compare only strings having the same length (L). The similarity score (S) is calculated as follow:

S=(L−D)/L (2)

#### Automatic resize

Because of the arbitrary selection of the initial start site, in some situations, the subject length may be different than the query one, but this could be corrected by selecting another start site. The “resize” function aims at solving this question. There are two distinct situations: (i) the query length is larger than the subject length; (ii) the query length is smaller.

**Fig 4:**
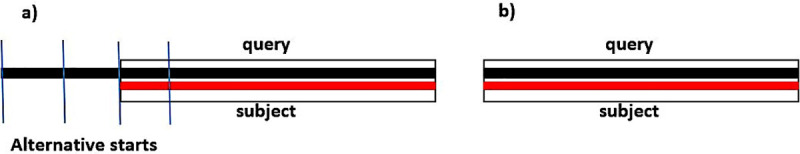
Identification of codons. Identification process of possible start codons in the genomic sequence and comparison to the information in the database; (a) alternative start sites and (b) result after adjustments.

In the first case, if the subject matches entirely with the query, but the query is larger than the subject, the tool looks for an alternative start site (valine, leucine or methionine) that reduces the query length to be closer to the subject (Fig 4).

**Fig 5:**
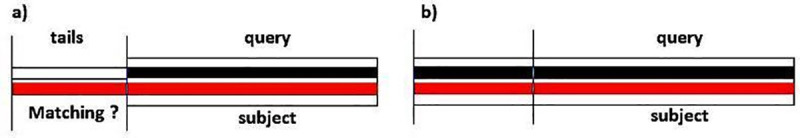
Codon Settings. Start codon adjustment process based on the information in the database; (a) analysis between query and subject, (b) result after adjustments.

In the second case (if the subject matches entirely with the query, but the subject is larger than the query) a start site further into the sequence (valine, leucine or methionine) is searched for before the query. However, there is no guarantee that the tail of the query matches with the tail of the subject. To confirm that both tails are lined up together, a first possibility would be to re-blast the query including its tail, whereas blasting has a high cost. To save this blast, we compare the tails similarity score. Then, if an alternative start can make the length of the ORF closer to the subject length, if the tails have a similarity score up to 90%, and if the query tail does not contain a stop codon, the ORF start site is replaced using the alternative start site (Fig 5).

**Fig 6:**
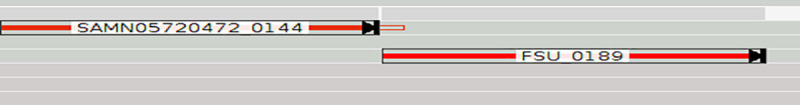
Visual representation for unmatching stop. The registered product with the ID SAMN05720472_0144 predicts a stop ahead compared to the query ORF length. Whereas the product FSU 0189 in the following ORF demonstrates a perfect length match between query and subject, in addition to having a high identity value.

In some circumstances, the head of the query or the head of the subject may be ahead of one compared to another. It means that the stop of the ORF and the stop of the deposited product (materialized by the subject) do not match. Nothing is done automatically to resolve the problem which means that there is no search for an alternative stop, whereas the graphical representation of the result points out it (Fig 6).

#### Blasting strategy

CODON enables a brute force strategy of automatically blasting every candidate ORF. While CODON is able to simultaneously perform several blasts, this strategy is timely as each blast takes between 1 and 5 minutes and many ORFs in overlapping regions may be blasted unnecessarily, increasing the process duration.

**Fig 7:**
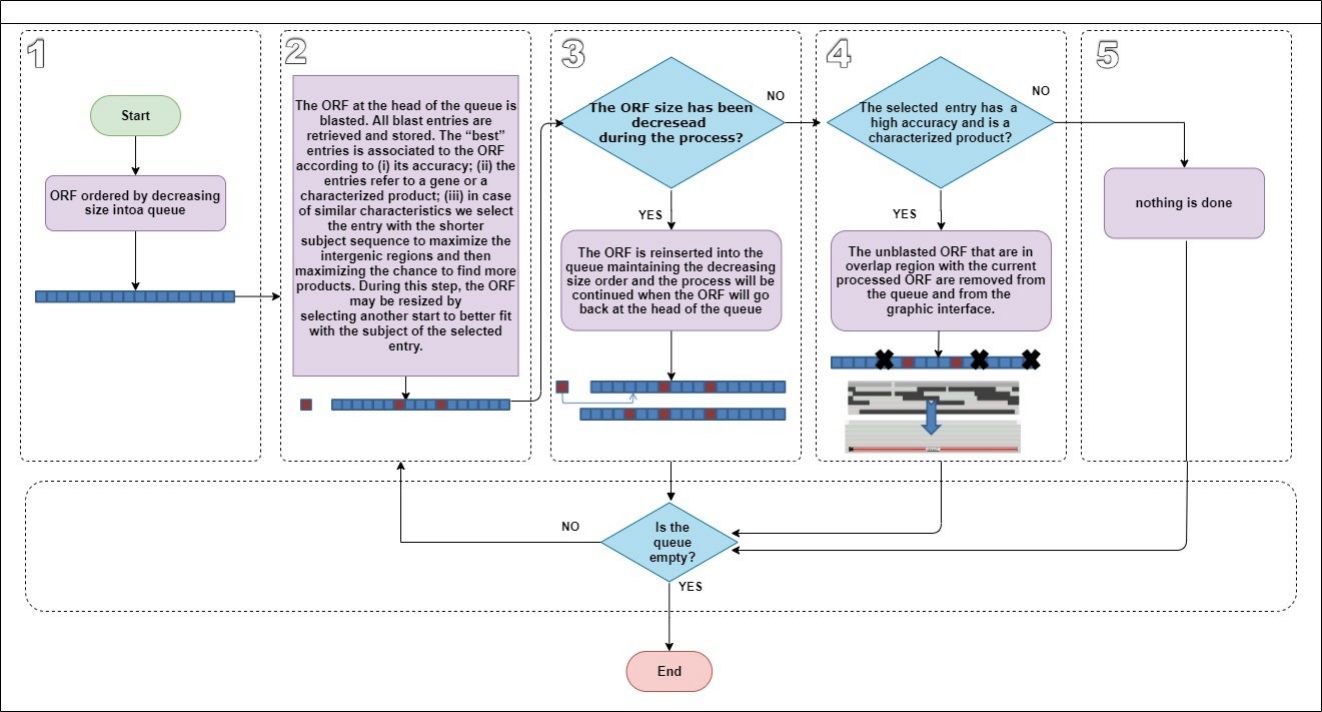
Blasting strategy. The optimized blast strategy algorithm tasks.

To save time, the annotation process implements an optimization strategy that consists of: (i) prioritizing the longer ORF as possible genes/products; (ii) cleaning candidate ORFs that are in overlapping areas with the recognized products that have a high accuracy; (iii) continuing to search for other products in the remaining intergenic regions. The algorithm is described (Fig 7).

The candidate ORFs (Fig 7 - Step 1) are ordered by decreasing length in a queue. When the blast of the first ORF in the queue result returns, the algorithm checks if a resize is possible (respecting the rules defined in the previous section), and calculates the accuracy of every entry with their eventual resized length. “MaxAcc” refers to the accuracy of the entry/entries with the highest accuracy and “Tolerance” to a threshold specified by the user. The default value for Tolerance is 2% (Fig 7 - Step 2).

The entries are ordered according to the following criteria: (i) if an entry has an accuracy up to (MaxAcc–Tolerance) and other entries have an accuracy below it, the first entry is preferred; (ii) if both entries have a similar accuracy then, if one entry refers to a gene and the other does not, the entry that refers to a gene is preferred; (iii) if both entries have a similar accuracy and both are not genes, if one has a product name and the other is a hypothetical protein, the entry that has a product name is preferred; (iv) if both entries have an accuracy value close and both are genes or both are products or both are hypotheticals, the entry with the better accuracy is preferred; (v) if the accuracy between the two entries are equal, the entry with the shorter matching sequence is preferred; and (vi) if the two entries have the same accuracy and length (they are considered as equals), the first one is preferred. The best entry is associated with the ORF and is eventually resized (Fig 7 - Step 2).

If the ORF size has been reduced by the resize and is now shorter than the next ORF in the queue, the ORF is reinserted into the waiting queue by order of length (Fig 7 - Step 3). When the ORF returns to the start of the queue, the first part of the process (blast and entry selection) will be ignored and the second part of the process (cleaning the other ORFs in overlapping areas with this ORF) will begin. If the ORF length remains unchanged or turned longer by the resize process, the second part of the process begins immediately (Fig 7 - Step 4).

If the blasted ORF (BORF) is a hypothetical protein or has an accuracy score lower than a threshold specified by the user (default value tool: 90%), the algorithm ignores this ORF and processes the next ORF in the queue. As a result, the other ORF in the overlapping area with the BORF will also be blasted to look for a possible characterized product with a higher accuracy. In the case where a characterized product was found with high accuracy, the algorithm will remove the overlaps between this ORF and the other ORFs that have not been blasted (OORF) present in the other frames by resizing them when possible or removing them (Fig 7 - Step 4).

**Fig 8:**
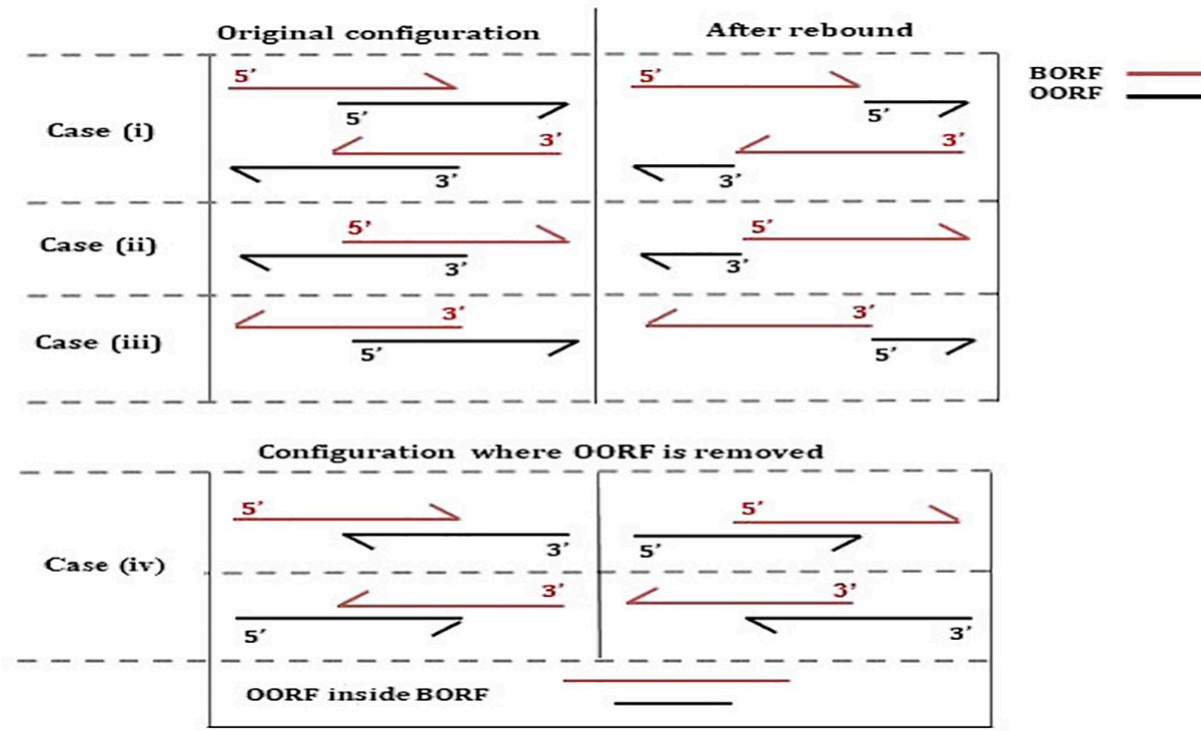
Filter strategies applied to the initial overlap solution. The cases require: BORF is a characterized product, BORF has an accuracy up to 90%, SBORF query and matching sequences fit together on their entire length, and OORF has never been processed.

If the OORF is blasted and has an accuracy lower than a threshold specified by the user (default value tool: 80%), the OORF is removed and no further processing is performed. If the OORF has never been blasted, different cases are possible: (i) if the BORF and OORF are both in forward frames and the BORF stop codon is before the OORF stop codon or if the BORF and OORF are both in reverse frames and the OORF stop codon is before the BORF stop codon (Fig 8 –case i), the algorithm will attempt to resize the OORF by using the first start codon in the OORF frame present between the BORF stop and the OORF stop. If there is no such start codon or if the new start codon makes the OORF very short (<60 bases), the OORF is removed; (ii) if the BORF is in a forward frame and the OORF in a reverse frame and the OORF stop codon is before the BORF start codon (Fig 8 –case ii), the algorithm attempts to resize the OORF by using the first start codon in the OORF frame present between the OORF stop codon and the BORF start codon; (iii) if the BORF is in a reverse frame and the OORF in a forward frame and the OORF start codon is after the BORF stop codon (Fig 8 –case iii), the algorithm will attempt to resize the OORF by using the first start codon in the OORF frame present between the BORF start codon and the OORF stop codon; (iv) if the overlap is up to a threshold specified by the user (default value tool) of the OORF length, the OORF frame is removed and further processing terminates (Fig 8 –case iv).

#### Pre-curation and annotation result improvement

**Fig 9:**
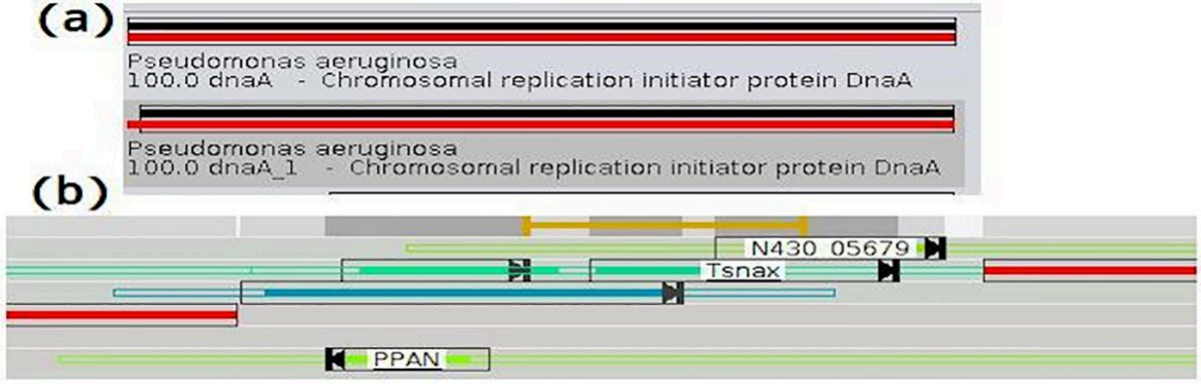
Blasting strategy on pre-curation. BLAST’s initial strategy to increase the possibility of identifying more gene products. In figure (a), a product with a high identity value is identified and initially the one with the shortest length is chosen. If the identified products do not have significant identity and accuracy value, they will be removed in the pre-curation stage, for example, N430 05679, Tsnax and PPAN (b).

The blasting strategy aims at increasing the intergenic regions by selecting the entry with the shorter subject size when two entries have equivalent characteristics. For instance, on the image capture (Fig 9A), both entries point out a gene (dnaA and dnaA_1) and both have high accuracy, then the first and shorter one will be initially selected. By maximizing the intergenic regions, the algorithm increases the probability to find out more products into these regions, but it also increases the number of blasted ORFs that have no relevant entry and would be discarded during a manual curation (Fig 9B).

CODON software offers the following pre-curation features, which enables to correct some problems and optimize the annotation before a manual curation.

**Fig 10:**
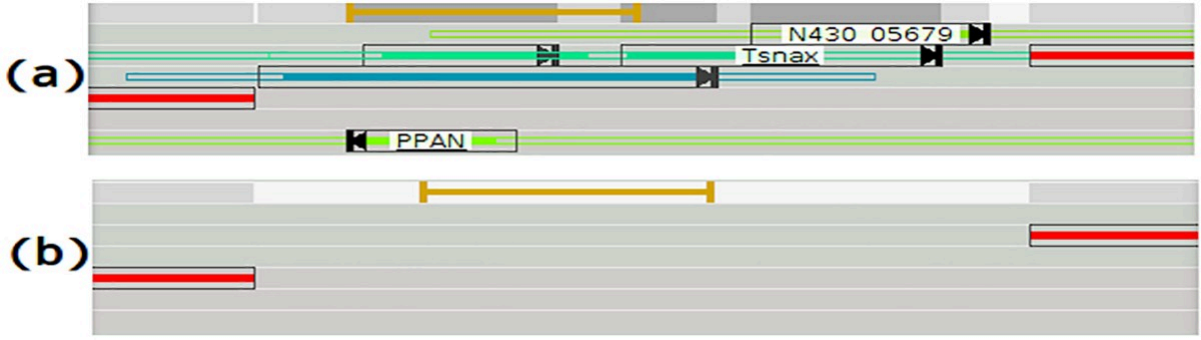
Low accuracy filter. Irrelevant ORF removed by low accuracy filter. (a) shows the products with an accuracy value below the value determined by the user. After the application of the filter, the products are removed (b).

***Low accuracy filter*.** The low accuracy filter enables the user to remove every ORF with an accuracy below a threshold value, removing ORFs with an accuracy of non-significant value. This parameter can be adjusted by the user in the graphical interface (Fig 10).

**Fig 11:**
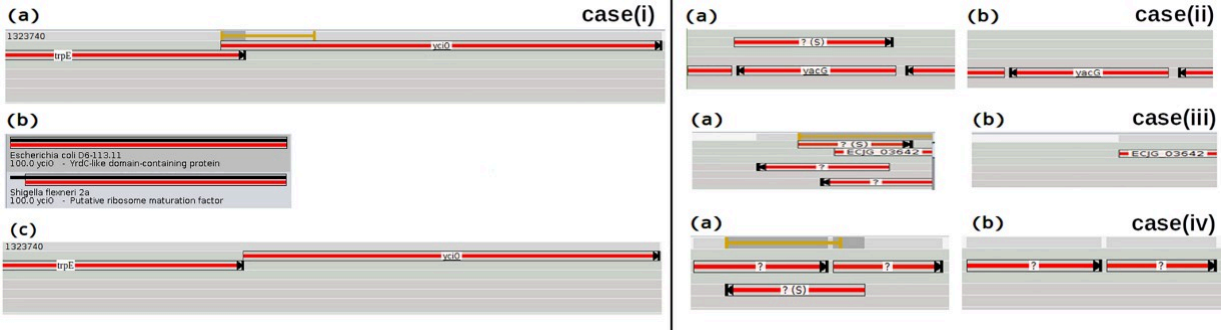
Overlap filter case(i). (a) The yciO gene is in an overlap region with the trpE gene. (b) There are several alternative entries for yciO. The algorithm removes the overlap by selecting the second entry that has a shorter size. (c) shows the ORF´s with the overlay resolved. **Overlap filter case(ii).** The Uncharacterized Protein in overlap region with the yacG gene is removed. **Overlap filter case(iii).** The Uncharacterized ORFs in overlap region with the protein ECJG_03642 are removed. **Overlap filter case(iv).** The Uncharacterized ORF in overlap region with two other ORFs is removed.

***Overlap filter*.** The overlap filter is divided into two steps. In the first step, the algorithm tries to resolve the overlap by selecting alternative entries with shorter products as illustrated in Fig 11 (to be selectable, an entry must also have an accuracy close to the best entry accuracy). It enables to select an alternative start and to reduce the ORF sizes so as to eliminate the overlap.

In case the overlap isn’t resolved in the first step, then the second step is initiated. It consists of removing on ORF into the overlap region, when possible, according to the following criteria (ordered by criterion priority): (i) if an ORF has an accuracy significantly higher than the other (difference lower than a threshold specified by the user), the ORF with the lower accuracy is removed; (ii) case an ORF has a gene acronym and the other is not, the other is removed (Fig 11); (iii) if an ORF is a characterized product and the other is not, the other is removed (Fig 11). The last observed case is when an uncharacterized ORF causes an overlap between two other ORFs, in which case it is removed (Fig 11).

**Fig 12:**
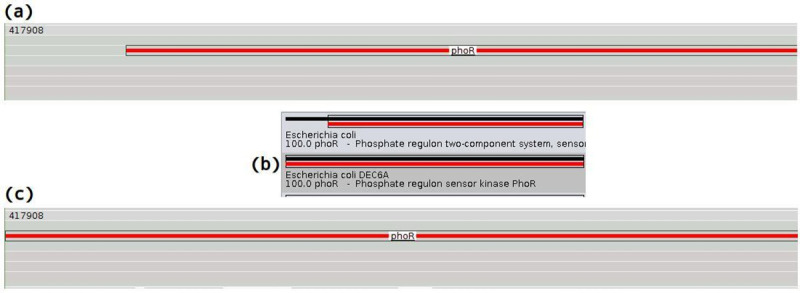
Reduction of intergenic region algorithm. In the initial condition, an intergenic region precedes the gene phoR (a), whereas the second entry has also a high accuracy, is also a gene, and has a longer subject, as shown in figure (b). The algorithm selects the second entry that better fulfills the intergenic region (c).

***Reduction of intergenic region algorithm*.** This algorithm aims at minimizing intergenic regions by expanding the remaining ORFs when possible. If two entries have characteristics equivalent, for example, close accuracy, both are gene or both are characterized protein whereas the subject has a different size. The algorithm will try to select the entry that better fulfills the intergenic region, without creating a new overlap (Fig 12).

### Similarity analysis

To perform the similarity analysis process, the BLAST version 2 [12] tool was used with the following parameters: e-value, -a determines the number of processors, -v which displays the number of hits in the database and -b which determines the number of alignments to be displayed, the values for these parameters were: 1e-05, 4 and 7, respectively, to obtain the best alignment results.

### Tool validation

To validate the software, eleven organisms deposited in the NCBI database were used, listed in Table 1. The FASTA files of these strains were used to annotate on the RAST platform and CODON software.

**Table 1:** The organisms used to validate the tool available for download in the NCBI database.

Organisms names	Taxonomic path (Phylum; Class; Order; Family; Genus; Specie)	Accession Number
*Escherichia coli* str. K-12 substr. MG1655	Proteobacteria; Gammaproteobacteria; Enterobacterales; Enterobacteriaceae; *Escherichia; Escherichia coli*.	CP014225
*Escherichia coli* str. K-12 substr. MC4100	Proteobacteria; Gammaproteobacteria; Enterobacterales; Enterobacteriaceae; *Escherichia; Escherichia coli*.	HG738867
*Planctomycetes bacterium* CA11	Planctomycetes; Planctomycetia; Planctomycetales; Planctomycetacea; *Planctomycetes; Planctomycetes bacterium*.	NZ_CP036353
*Salmonella enterica* strain FDAARGOS_768	Proteobacteria; Gammaproteobacteria; Enterobacterales; Enterobacteriaceae; *Salmonella; Salmonella enterica*.	NZ_CP041005
*Mycobacterium tuberculosis* H37Rv	Actinobacteria; Actinobacteria; Corynebacteriales; Mycobacteriaceae; *Mycobacterium; Mycobacterium tuberculosis*.	AL123456 BX842572-BX842584
*Klebsiella variicola* strain KP5-1	Proteobacteria; Gammaproteobacteria; Enterobacterales; Enterobacteriaceae; *Klebsiella; Klebsiella variicola*	CP008700
*Streptococcus pneumoniae* strain M26365	Firmicutes; Bacilli; Lactobacillales; Streptococcaceae; *Streptococcus; Streptococcus pneumonia*.	NZ_CP031248
*Fibrobacter succinogenes* subsp. succinogenes S85	Fibrobacteres; Fibrobacteria; Fibrobacterales; Fibrobacteraceae; *Fibrobacter; Fibrobacter succinogenes*.	CP002158
*Vibrio parahaemolyticus* BB22OP	Proteobacteria; Gammaproteobacteria; Vibrionales; Vibrionaceae; *Vibrio; Vibrio parahaemolyticus*.	NC_019955
*Nocardia farcinica* strain NCTC11134	Actinobacteria; Actinobacteria; Corynebacteriales; Nocardiaceae; *Nocardia; Nocardia farcinica*	NZ_LN868942
*Pseudomonas aeruginosa* LESB58	Proteobacteria; Gammaproteobacteria; Pseudomonadales; Pseudomonadaceae; *Pseudomonas; Pseudomonas aeruginosa*.	NC_011770

The CODON EMBL is generated from the same FASTA file by the CODON prediction and annotation process. CODON marked the hypothetical ORFs, performed the optimized blast strategy, passed the information through the low accuracy filter with a threshold of 80%, removed the overlaps when possible and maximized the intergenic region occupation through the last filter. The result was exported as an EMBL file.

The NCBI annotation was improved by CODON. The NCBI EMBL file was loaded into CODON. The NCBI predicted ORFs were all blasted and the filters were applied to generate the CODON EMBL files (the ORFs with a low accuracy were removed, the overlaps were removed when possible and the occupation of intergenic regions was maximized). The same process was performed to generate the RAST EMBL with high annotation accuracy.

## Results

The prediction and annotation results using the CODON software for all organisms were compared with information from the RAST platform as well as the strain information deposited in the NCBI database. Table 2 shows the amount of products present in the annotations of NCBI, RAST and CODON.

**Table 2:** Total quantity of products present in the NCBI, RAST and CODON annotations.

Organism	NCBI	RAST	CODON
*Escherichia coli* str. K-12 substr. MG1655	4488	4557	5405
*Escherichia coli* str. K-12 substr. MC4100	4025	4415	5352
*Planctomycetes bacterium* CA11	5701	6530	6180
*Salmonella enterica* strain FDAARGOS_768	4566	4850	5406
*Mycobacterium tuberculosis* H37Rv	4030	4299	4852
*Klebsiella variicola* strain KP5-1	4755	5314	5578
*Streptococcus pneumoniae* strain M26365	2164	2295	2545
*Fibrobacter succinogenes* subsp. succinogenes S85	2871	3305	3056
*Vibrio parahaemolyticus* BB22OP	2925	2998	3087
*Nocardia farcinica* strain NCTC11134	3426	3546	3581
*Pseudomonas aeruginosa* LESB58	6088	6251	6388

Table 3 shows the similarity analysis using the BLAST version 3 tool with the information deposited at the NCBI as a control. Based on the match percentage returned by the BLAST, the products that obtained a match of one hundred percent and those that obtained a match between sixty and below one hundred percent were counted. In this analysis, the coding sequences (CDS) extracted from the files in the EMBL format containing the CODON and RAST annotations were used as input.

**Table 3:** BLAST similarity analysis between the CDS extracted from each EMBL file.

Organism^a^	Tool^b^	Match 100%^c^	Match between 60% and 100%^d^
*Escherichia coli* str. K-12 substr. MG1655	RAST	4406	16
	CODON	4607	128
*Escherichia coli* str. K-12 substr. MC4100	RAST	4026	45
	CODON	4148	59
*Planctomycetes bacterium* CA11	RAST	5727	86
	CODON	5669	59
*Salmonella enterica* strain FDAARGOS_768	RAST	4542	16
	CODON	4564	48
*Mycobacterium tuberculosis* H37Rv	RAST	4042	32
	CODON	4768	46
*Klebsiella variicola* strain KP5-1	RAST	4739	66
	CODON	4768	37
*Streptococcus pneumoniae* strain M26365	RAST	2184	48
	CODON	2213	129
*Fibrobacter succinogenes* subsp. succinogenes S85	RAST	2876	55
	CODON	2854	26
*Vibrio parahaemolyticus* BB22OP	RAST	2886	11
	CODON	2870	8
*Nocardia farcinica* strain NCTC11134	RAST	3414	6
	CODON	3381	3
*Pseudomonas aeruginosa* LESB58	RAST	6076	5
	CODON	6124	5

a Column 1 (Organism name) shows the name of the organism.

b Column 2 (Tool) the tools used for the annotation process.

^c^ Column 3 (Match 100%) shows the amount of products that showed 100% similarity and

^d^ Column 4 (Match between 60% and 100%) shows the amount products that are in the range between 60% to 100% that have an alignment length equal to the size of the CDS.

With the analysis of the data presented in Table 3, it is possible to observe that despite the annotation performed by CODON not suffered any manual curation process, in many cases, it presents a high similarity between the products annotated by CODON with those of NCBI present in the local BLAST database.

Initially, the total number of products with a gene acronym was only possible to calculate in the EMBL files of NCBI and CODON given that RAST does not provide a gene acronym in the annotated CDS. To improve and try to remedy the previous problem toward annotation from RAST, the EMBL files of RAST and NCBI were submitted to the re-annotation process using the CODON software and the product with a gene acronym calculated from the re-annotation result. The result is listed in Table 4.

**Table 4:** Displays the amount of products with gene acronym results obtained with the re-annotation process using the CODON software.

Organism	NCBI	CODON	NCBI re-annotation	RAST re-annotation
*Escherichia coli* str. K-12 substr. MG1655	645	4233	4150	4181
*Escherichia coli* str. K-12 substr. MC4100	5079	4140	3991	4117
*Planctomycetes bacterium* CA11	500	2229	2044	2004
*Salmonella enterica* strain FDAARGOS_768	2270	3979	3973	3999
*Mycobacterium tuberculosis* H37Rv	2033	2424	2219	2098
*Klebsiella variicola* strain KP5-1	562	4186	4050	4188
*Streptococcus pneumoniae* strain M26365	447	1479	1431	1452
*Fibrobacter succinogenes* subsp. succinogenes S85	568	607	569	678
*Vibrio parahaemolyticus* BB22OP	1098	2427	1428	1416
*Nocardia farcinica* strain NCTC11134	312	1738	1554	1653
*Pseudomonas aeruginosa* LESB58	1285	4205	4055	4107

The use of CODON increased the number of products annotated with gene acronym in the NCBI and RAST EMBL files, and the number of genes annotated in the NCBI analysis was significantly increased in comparison to the annotations present in the previous EMBL file. However, it can be observed that the organisms annotated with CODON presented the largest number of products with a gene acronym, this is due to the annotation process used in the CODON software.

The annotation quality can be measured by the average accuracy (the accuracy calculation is described in the methodology section item Algorithms and Metrics) calculated by CODON for all annotated products. It can be checked directly in the CODON interface in the reports menu. For every EMBL generated in this study, the average accuracy varies from 98.85 to 99.87 proving a deep similarity between the product annotated and the product deposited in the UNIPROT databases. The analysis of the number of hypothetical proteins present in each EMBL file is listed in Table 5.

**Table 5:** Amount of hypothetical proteins present in the NCBI, RAST, CODON annotations and in the EMBL files generated after the re-annotation process.

Organism	CODON	RAST	NCBI	RAST re-annotation	NCBI re-annotation
*Escherichia coli* str. K-12 substr. MG1655	940	269	1113	171	120
*Escherichia coli* str. K-12 substr. MC4100	662	255	716	181	4
*Planctomycetes bacterium* CA11	2077	4226	1484	2046	1732
*Salmonella enterica* strain FDAARGOS_768	950	531	211	334	37
*Mycobacterium tuberculosis* H37Rv	843	872	648	351	238
*Klebsiella variicola* strain KP5-1	803	629	705	313	170
*Streptococcus pneumoniae* strain M26365	368	455	255	183	118
*Fibrobacter succinogenes* subsp. succinogenes S85	664	1536	830	586	636
*Vibrio parahaemolyticus* BB22OP	237	539	265	163	97
*Nocardia farcinica* strain NCTC11134	557	1046	676	569	553
*Pseudomonas aeruginosa* LESB58	740	1243	873	624	535

The analysis of the number of hypothetical proteins described in Table 5 showed that in some cases there was a greater number in the CODON annotation, which emphasizes the need for a manual curation process with the next step. However, in some cases, these additional hypothetical proteins fulfilling intergenic regions, match with hypothetical proteins deposited in the UNIPROT database with high accuracies. It is also possible to observe that there was a reduction in the number of hypothetical proteins after the re-annotation process of the NCBI and RAST EMBL files.

Another important feature that CODON has, beyond the addition of the gene acronym during annotation, is the identification of transfer ribonucleic acid (tRNA), and ribosomal RNA (rRNA). The results of this analysis can be seen in Tables 6 and 7, respectively.

**Table 6:** Amount of tRNA predicted on NCBI, RAST, and CODON annotation for each organism.

Organism	NCBI	RAST	CODON
*Escherichia coli* str. K-12 substr. MG1655	87	88	112
*Escherichia coli* str. K-12 substr. MC4100	88	87	112
*Planctomycetes bacterium* CA11	57	58	66
*Salmonella enterica* strain FDAARGOS_768	86	86	100
*Mycobacterium tuberculosis* H37Rv	45	44	55
*Klebsiella variicola* strain KP5-1	77	76	83
*Streptococcus pneumoniae* strain M26365	58	58	57
*Fibrobacter succinogenes* subsp. succinogenes S85	58	57	58
*Vibrio parahaemolyticus* BB22OP	112	112	142
*Nocardia farcinica* strain NCTC11134	22	22	26
*Pseudomonas aeruginosa* LESB58	67	68	85

**Table 7:** Amount of rRNA predicted on NCBI, RAST, and CODON annotation for each organism.

Organism	NCBI	RAST	CODON
*Escherichia coli* str. K-12 substr. MG1655	22	22	24
*Escherichia coli* str. K-12 substr. MC4100	22	22	24
*Planctomycetes bacterium* CA11	4	4	4
*Salmonella enterica* strain FDAARGOS_768	22	22	22
*Mycobacterium tuberculosis* H37Rv	3	3	3
*Klebsiella variicola* strain KP5-1	5	5	5
*Streptococcus pneumoniae* strain M26365	12	12	12
*Fibrobacter succinogenes* subsp. succinogenes S85	9	9	9
*Vibrio parahaemolyticus* BB22OP	31	31	31
*Nocardia farcinica* strain NCTC11134	9	9	8
*Pseudomonas aeruginosa* LESB58	12	12	18

The CODON software incorporates the prediction of these ribosomes in the analysis stage by searching for similarity in the tRNAscan-SE and RNAmmer databases. Thus, the user does not need to perform the prediction of these elements manually, as described in the section Prediction and annotation of tRNAs and rRNAs.

The results described in Tables 6 and 7 demonstrate that even without the manual curation process in several cases the amount of tRNA and rRNA identified in the annotation performed by CODON are the same as those observed in the annotations of NCBI and RAST.

**Fig 13:**
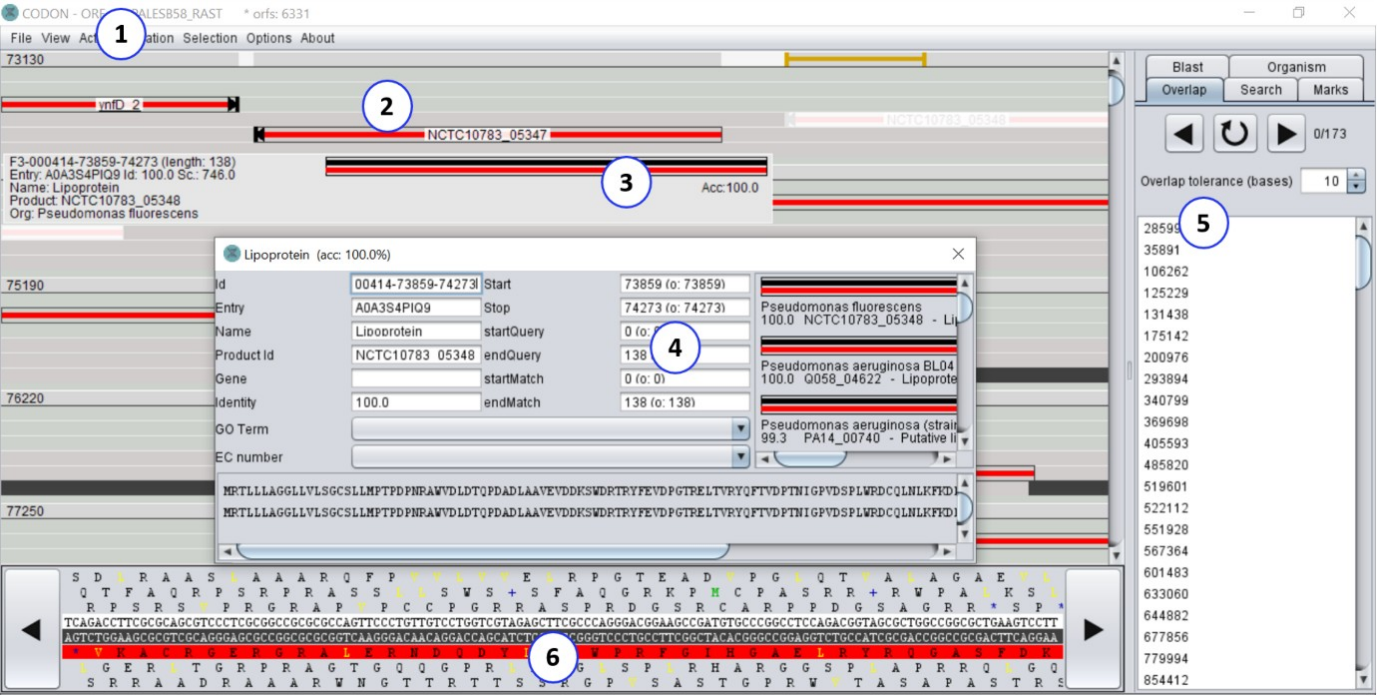
CODON Overview. (1) Main Menu, shows all options available in the tool; (2) Main view, displays a compact result of the prediction and annotation process. Every predicted ORF can be analyzed; (3) ORF Annotation Information, displays basic information about the ORF (product name, acronym gene, organism entry math, and percentage of identity) when the ORF is selected by a click; (4) ORF Details, displays more details about the ORF and enables to explore deeper the annotation result. To view this option, double click on the ORF; (5) Side bar, enables to monitor the annotation task progression and to explore the results; (6) Sequence details view enables to analyze accurately specifics subsequences and to edit the sequence.

In addition to these advantages, CODON allows manual curation through the graphical interface, which the user can select in the Uniprot database hits ordered by accuracy (Fig 13). In this way, the user will be able to analyze the CDS based on identity and length information of the mapping according to the aligned hits. Thus, it enables the user to adjust the CDS without having to perform external alignment of each CDS.

All the functions implemented in the CODON software aimed at reducing the work to perform the manual curation by bringing all information necessary to analyse every product and the graphical function to edit it.

Based on these results, CODON is an efficient tool for ORF prediction and annotation. The bases used to search for information on annotations proved to be highly cured considering the number of products with gene acronyms present in the EMBL file generated from CODON. The predicted products were length-adjusted according to the metrics of accuracy and identity in comparison with the results obtained in the other databases.

**Fig 14:**
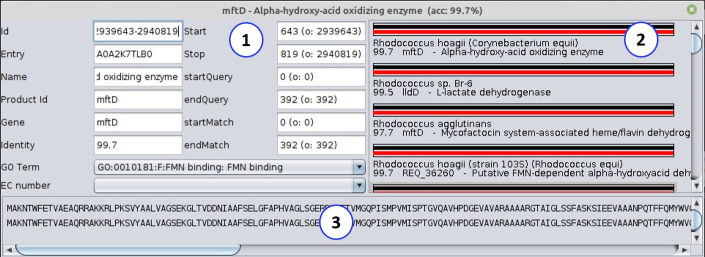
Detailed annotation. Display the screen with detailed annotation beyond all gene products that showed similarity in the database, among the information, there is, percentage of identity, product name, gene acronym if any, and the name of the organism. (1) Displays the detailed information about the ORF; (2) Demonstrates all the retrieved entries in the Uniprot database that matched the ORF and (3) displays the query and subject sequences.

By using the EMBL files from RAST and NCBI in CODON, it was possible to improve the annotation with the identification of additional genes and to reduce the number of hypothetical proteins resulting in more accurate products as highlighted by the accuracy averages. The graphic interface of CODON facilitates the manual curation process (Fig 14), considering that the user can use it to verify each annotated product and identify the top hits found in the databases for a blasted ORF. It is worth mentioning that each hit is accompanied by information such as percentage of identity (which is also shown graphically so that the user can assess the length of the alignment), query and subject subsequences that match together, the name of the gene or product, and the organism in which that product was found in the database, resulting in a greater wealth of details compared to other annotation.

Table 8 highlights some functions performed by CODON in the comparison between RAST and ARTEMIS, one very popular to perform the annotation process in prokaryotic genomes and the other widely used in the process of visualizing information on gene products, as well as in manual curation.

**Table 8:** Functions by tool. Summary about tasks performed by software RAST, Artemis and CODON.

Tasks description	RAST	Artemis	CODON
Annotation	ok	-	ok
Manual curation	-	ok	ok
Visualize the genes products	-	ok	ok
Edit the CDS informations	-	ok	ok
Easy editing of CDS information directly in the graphical interface, based on the entries obtained in the Uniprot database	-	-	ok
Has metrics to minimize intergenic areas	-	-	ok
The annotation process can be divided between several curators, in the end the tasks can be consolidated into a single project.	-	-	ok
Direct visualization of identity and accuracy percentages, and subject and query matches areas			ok

Despite performing the annotation process as well as the Web RAST platform, the CODON software uses a different strategy. The ORFs are identified using a finite state machine and CODON uses the nucleotide sequences of these ORFS to search for similarity in the UniProt database retrieving all products that obtained a match with the submitted sequence. This information is used to feed the user who performs manual curation with an environment that concentrates all information with greater accuracy, since Uniprot is considered a highly accurate database, in a simple and intuitive graphical interface.

The metrics described in the manuscript were implemented to assist in the process of better adjustment of the information to be displayed to the user. In addition it makes automatic adjustments, for example, treatment of overlap between products, adjustment for the best match between query and subject, reduction of the intergenic zone with the automated definition for start codon (based on match obtained in the Uniprot database).

The results presented in this analysis prove the efficiency of the annotation process performed in CODON software. However, the main focus of CODON is to be a tool to assist in the manual curation process, since the user can perform the annotation process using any other software and submit this result as input to CODON. It is worth noting that RAST is not used as a manual curation platform, but the results are used in manual curation using the ARTEMIS software.

During the task of annotation enrichment (re-annotation), all coordinates of the ORFs predicted by another platform are used by CODON. Then, CODON continues the workflow annotation as described above. At the end of the curation process the user can export his new file in EMBL format or in a software format called the CODON project. It is concluded that the CODON software concentrates two functions, annotation and manual curation. In addition, it facilitates the manual curation process since the user does not need to consult external bases to prove identity values, alignment length or even if the predicted product is similar in other organisms. These external consults must be done when doing manual curation using ARTEMIS and the results of these consults must be manually inserted into the ARTEMIS graphical interface.

All the match information obtained between the query sequence and the Uniprot database are retrieved and saved by ORF in the project folder, all alignment information obtained between query and subject are displayed in the CODON interface, however, the automatic choice of the information displayed is based primarily on the best match which has the highest identity value between query and subject and on the metrics implemented in the software and already described in the manuscript.

The interface allows the user to select a particular ORF, view the information in detail, perform editing by choosing between all the products retrieved from the database that were matched with the ORF, as described in Fig 1 item 04, after adjustments the information is updated in the CODON interface and saved for later exportation of the results.

Thus, it can be concluded that the CODON software is a viable alternative to carry out the manual curation process in addition to presenting a new strategy to perform the annotation process for prokaryotic genomes.

### Availability and future directions

The CODON software is available in https://sourceforge.net/projects/codon-software. For future work, in order to enrich the information provided by CODON, a new feature that is being studied for implementation in a future version of CODON is the annotation of mobile genetic elements (MGEs). These elements are related to evolution, adaptation, virulence and resistance to antibiotics and heavy metals. The annotation of MGEs present in the analyzed genomes will be interesting for the community as it will create important information and, to date, few tools are able to produce.

Currently, there are some databases and programs that present specific information about MGEs such as: ISfinder, ISEScan, ACLAME, MGERT, ICEberg, MGEfinder, ISMapper, among others. The objective is to analyze these different sources of information and integrate this data with CODON, thus allowing the CODON annotation and curation process to be able to identify and annotate MGEs automatically, just as CODON does with coding sequences.
